# Demographic Processes Drive Increases in Wildlife Disease following Population Reduction

**DOI:** 10.1371/journal.pone.0086563

**Published:** 2014-05-02

**Authors:** Jamie C. Prentice, Glenn Marion, Piran C. L. White, Ross S. Davidson, Michael R. Hutchings

**Affiliations:** 1 Disease Systems Team, SRUC, Edinburgh, United Kingdom; 2 Biomathematics and Statistics Scotland, Edinburgh, United Kingdom; 3 Environment Department, University of York, York, United Kingdom; University of Oxford, Viet Nam

## Abstract

Population reduction is often used as a control strategy when managing infectious diseases in wildlife populations in order to reduce host density below a critical threshold. However, population reduction can disrupt existing social and demographic structures leading to changes in observed host behaviour that may result in enhanced disease transmission. Such effects have been observed in several disease systems, notably badgers and bovine tuberculosis. Here we characterise the fundamental properties of disease systems for which such effects undermine the disease control benefits of population reduction.

By quantifying the size of response to population reduction in terms of enhanced transmission within a generic non-spatial model, the properties of disease systems in which such effects reduce or even reverse the disease control benefits of population reduction are identified. If population reduction is not sufficiently severe, then enhanced transmission can lead to the counter intuitive perturbation effect, whereby disease levels increase or persist where they would otherwise die out. Perturbation effects are largest for systems with low levels of disease, e.g. low levels of endemicity or emerging disease.

Analysis of a stochastic spatial meta-population model of demography and disease dynamics leads to qualitatively similar conclusions. Moreover, enhanced transmission itself is found to arise as an emergent property of density dependent dispersal in such systems. This spatial analysis also shows that, below some threshold, population reduction can rapidly increase the area affected by disease, potentially expanding risks to sympatric species.

Our results suggest that the impact of population reduction on social and demographic structures is likely to undermine disease control in many systems, and in severe cases leads to the perturbation effect. Social and demographic mechanisms that enhance transmission following population reduction should therefore be routinely considered when designing control programmes.

## Introduction

The relevance of ecology to understanding the dynamics and persistence of infectious disease has long been recognised [1], and ecological factors are critical to wildlife disease systems. Control of disease in wildlife is of considerable importance for managing risks to humans [2], [3] and livestock [4], [5], as well as for the conservation of wildlife species themselves [3], [6]–[8]. Population reduction is a commonly employed strategy used to control disease in wildlife [9], [10] with the aim of reducing the number of infected animals and the overall size of key populations, leading to a reduction in rates of transmission, disease prevalence and risks to other populations. Application of this strategy is supported by theoretical evidence of a threshold for disease persistence below which disease does not spread quickly enough to persist, and eventually dies out [9], [11], [12]. However, there is growing evidence that population reduction may be less effective than standard analyses predict, and in some cases be counter-productive (see below). Such unexpected increases in disease prevalence following population reduction have been termed the “perturbation effect” [13]. The theoretical basis and empirical evidence for disease thresholds in wildlife has been reviewed [14], concluding that important elements of wildlife ecology are neglected by current theories.

It is known that the social and spatial structure of host populations has significant implications for disease persistence and prevalence [15], [16]. Population reduction disrupts existing social structures and this may lead to increased numbers of contacts [17] and/or a greater proportion of agonistic encounters within or between groups [18], [19]. Similarly, a change in susceptibility of individual hosts may also occur as a consequence of population reduction due to stress [20]. Both effects will enhance disease transmission and are likely to be widespread and reduce or even reverse the efficacy of population reduction measures.

For example, management of rabies in foxes (*Vulpes vulpes*) has shown that vaccination is more suitable than culling, as the latter can destabilise social structure and lead to enhanced transmission rates [10], [21]. Studies of the management of classical swine fever (CSF) in wild boar (*Sus scrofa*) recommend that hunting should cease following detection of the disease [22], in order to discourage dispersal of infected individuals, and reduce risks to neighbouring groups [10]. The U.K. Randomised Badger Control Trial (RBCT) [23] showed that reactive culling of badgers (*Meles meles*) in response to a confirmed bovine tuberculosis (*Mycobacterium bovis*, bTB) herd breakdown in cattle, was associated with a 27% increase in the incidence of confirmed breakdowns, relative to survey-only trials [24]. Repeated reactive culling was also associated with increased bTB prevalence in badgers [25].

In this paper we study the potential for behavioural and demographic aspects of the ecology of wildlife species to reduce or reverse the efficacy of population reduction as a means of disease control. Our results are based on the analytical and numerical treatment of generic models of demography and disease dynamics in wildlife populations. In a non-spatial context we analyse the potential that individual and collective behavioural responses to population reduction have on disease control. We use this framework to explore the demographic and epidemiological characteristics of wildlife disease systems that make them susceptible to such effects. We then demonstrate that such impacts arise as an emergent property of spatial models of wildlife disease systems with density dependent dispersal. Finally we discuss the significance of these results for disease control in wildlife.

## Methods

### A non-spatial deterministic model of demography and disease dynamics

We examine a generic single pathogen wildlife disease system with a fluctuating host population. The number of susceptible and infected individuals in the population at time *t* are *S*(*t*) and *I*(*t*) respectively, and the total population size is given by *N*(*t*) = *S*(*t*)+*I*(*t*). We assume density dependent (logistic) growth, with intrinsic reproduction rate *r* (the maximum rate that individuals can reproduce in optimal circumstances), limited by a carrying capacity *c* (the population size for which the density limited per-capita birth rate reaches zero — note this is not necessarily the same as the population equilibrium [26], [27], since mortality, including that induced by disease and population reduction, will prevent the population from attaining this maximum). Natural mortality (from causes unrelated to disease or explicit population reduction measures) occurs at constant per-capita rate *d*, while disease induced mortality occurs at constant per-capita rate *e*. The rate of infection is a combination of susceptibility and contact rates between susceptible and infective individuals and here we consider density dependent infection (i.e. disease transmission depends on the density of infectives, *I*) with horizontal transmission rate *β*.

We model population reduction as a constant per-capita death rate *p* which applies to all individuals regardless of disease status. As noted earlier such measures can alter host behaviour and hence contact rates. We therefore model the horizontal disease transmission rate as *β*+*kp*. Here *k*>0 represents any mechanism or combination of mechanisms that lead to increased contact rates or susceptibility in a host population subjected to population reduction at rate *p*. Note that this formulation represents a simplification in that the effect is linear in *p*, there is no lag as *p* changes and the effect is constant for the duration of the population reduction event.

In Appendix S1 (see Supporting Information, File S1), we show how to formulate a simple non-spatial deterministic model that encapsulates the above assumptions. We also simplify this representation, removing the variables *c* and *r* by respectively scaling the variables *S*, *I* and *N* by 1/*c* to obtain values between 0 (empty) and 1 (at carrying capacity) and rescaling time by *r* (see Appendix S1.2, Eqn. S1 in File S1). Analysis can then focus on the effects of population characteristics (parameters *d* and *e*), disease dynamics (*β*), population reduction (*p*), enhanced transmission (*k*) and the interactions between them. However, results for specific values of *c* and *r* can still be obtained by appropriate back scaling. The rescaled deterministic ordinary differential equations (ODEs) that combine the demography and disease dynamics described above with population reduction and a corresponding enhanced transmission resulting from explicit behavioural and implicit ecological (system) responses are given by:
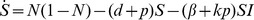
(1)

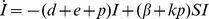
Three fixed points of this system of equations are derived in the Supporting Information: population extinction, where 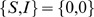; the disease free equilibrium, where 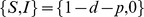; and the endemic equilibrium 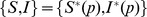, where both the population and the disease persist (note that it is possible for 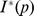 to be negative, in which case 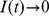, since there cannot be a negative number of individuals). The stability properties of these equilibria are discussed in Appendix S1.4 in File S1. Note that we write the endemic equilibrium as a function of the reduction rate *p*, even though it also depends on other parameters, because we are particularly interested in the effect of population reduction.

### A spatial stochastic model of demography and disease dynamics

In the stochastic spatial model we consider a set of sites where, at time *t*, the integer number of susceptibles and infectives in site *i* are 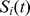 and 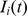 respectively. Since we are dealing with numbers of individuals these are not rescaled as above. The demography and disease dynamics of each sub-population are governed by the same processes as for the non-spatial model, with the addition of dispersal and disease transmission within and between groups.

Dispersal is the movement of individuals between social groups, for the purposes of obtaining more resources such as food or reproductive opportunities (including inbreeding avoidance). In the model dispersal from any given site occurs at constant per-capita rate *m*, into any of its nearest neighbouring sites. However, since this process may be mediated by the population levels in the destination site [28]–[30] this is modified by a function 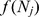, where *N_j_* is the population at neighbouring site *j*. We consider a step function
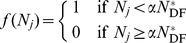
(2)where 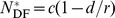 is the population size in the disease free equilibrium, and *α* is the fraction of the disease free equilibrium at which the neighbouring site becomes accessible. Dispersal rates may also be affected by conditions in the source area, e.g. due to overpopulation, social exclusion, or lack of resources, lack of mating opportunities in small populations; however, we do not consider these effects here.

Disease transmission rates within and between groups are denoted *β_w_* and *β_b_* respectively. The horizontal disease transmission rate in site *i* is therefore given by
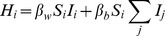
where the sum is over neighbouring sites of *i*. The total infection rate is given by 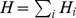 and the effective disease transmission rate is defined as
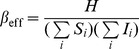
The spatial model is implemented as a discrete state-space Markov process, to account for demographic stochasticity, with events and associated rates shown in Table 1, and simulated using the Gillespie algorithm [31]. In the spatial model population reduction is parametrised by the probability that a site is targeted *p*_1_ and the rate of removal of individuals within targeted sites *p*_2_.

**Table 1:** Default event rates for the stochastic *SI* model.

Event	Rate	*δS_i_*	*δI_i_*	*δS_j_*	*δI_j_*
Birth of *S_i_*	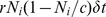	+1	0	0	0
Death of *S_i_*	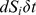	−1	0	0	0
Death of *I_i_*	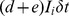	0	−1	0	0
Infection of *S_i_*	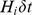	−1	+1	0	0
Dispersal of *S_i_* to site *j*	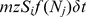	−1	0	+1	0
Dispersal of *I_i_* to site *j*	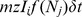	0	−1	0	+1

Event rates and corresponding effects in the spatial stochastic model. *H_i_* and *f*(*N_j_*) are defined in the methods.

**Table 2:** List of parameters used in the deterministic and stochastic *SI* models.

Parameter	Symbol	Non-spatial	Spatial
Intrinsic reproduction rate	*r*	1	1
Carrying capacity	*c*	1	20
Natural mortality rate	*d*	0.2	0.01
Disease induced mortality rate	*e*	0.1	0.1
Horizontal transmission rate	*β*	0.4	—
background	*β_e_*	—	0
within group	*β_w_*	—	0.5
between groups	*β_b_*	—	0
Dispersal rate	*m*	—	0.1
threshold value	*α*	—	0.7
Population reduction rate	*p*	0.1	—
coverage	*p*_1_	—	0.2
removal within sites	*p*_2_	—	0.5
Disease enhancement	*k*	5	—

A summary of the parameters and their symbols used in the non-spatial and spatial models are described here. Values shown indicate both the parameters and their default values used in the spatial and non-spatial models.

### Measuring the perturbation effect

We define the magnitude of the perturbation effect at time *t* after the application of population reduction at rate *p* to be
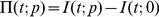
(3)


A population that is in equilibrium 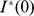 prior to the application of population reduction at rate *p*, will reach a new equilibrium 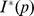. We define the persistent perturbation effect
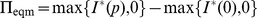
(4)


Note that 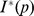 may be negative, in which case the equilibrium is no longer stable and cannot be reached, and so 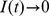, hence the restrictions (see Appendix S1.4 in File S1 for more details). In the results, we study both the persistent 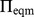 and transient 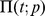 perturbation effects. In the spatial case we also examine the proportion of sites containing infectives, 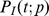, as the basis for measuring the perturbation effect
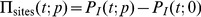
(5)


## Results

### Explicit enhancement of disease transmission induced by population reduction

**Figure 1:**
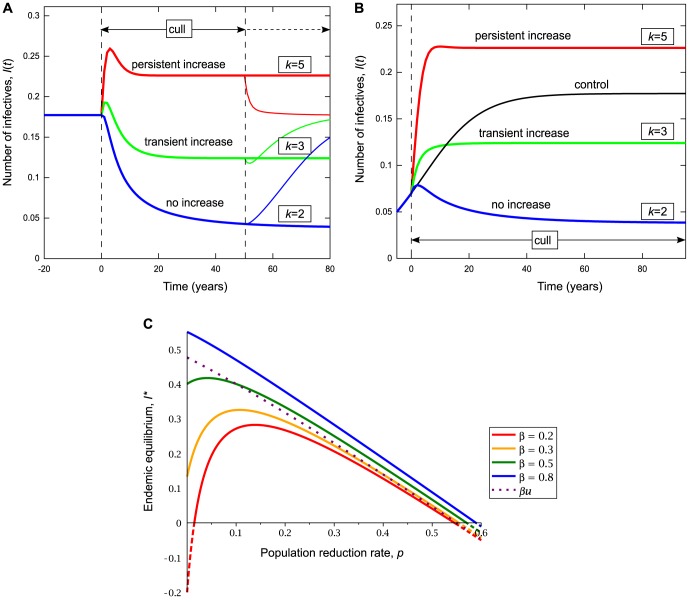
Deterministic simulation of *I*(*t*), and algebraic solution of I^*^(*p*). The results of ongoing population reduction are shown for various levels of disease enhancement *k* in (A) endemic disease, (B) emergent disease (starting near the disease free equilibrium, 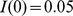). (C) shows the endemic equilibrium for varying *β*. The lines cut the vertical axis at 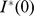, and so the perturbation effect occurs whenever a line rises above this value. Note that for *β* = 0.2, the equilibrium is negative for small *p* (which cannot be reached, since only a non-negative number of individuals is biologically possible), and so if any disease is introduced for *p* = 0, it moves to the disease free equilibrium 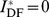, and the perturbation effect does not occur until *p* is sufficiently high. The dotted line shows *β_u_* (see text for details), marking the upper bound of *β* for given *p* for which the perturbation effect is possible, and crosses each line at the point where the increase no longer occurs for that value of *β* (*β_u_* is also illustrated in Fig. 2A). Parameters are given in Table 2, except *p* = 0.2 in (A) and (B).

We first consider the perturbation effect in the deterministic non-spatial model. Several features of the perturbation effect caused by increased horizontal disease transmission in response to population reduction are demonstrated in Fig. 1. For different levels of transmission enhancement *k*, a range of outcomes are possible when a population in the endemic equilibrium 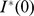 (disease endemic before intervention starts), is subjected to sustained population reduction at rate *p* (see Fig. 1A). The long term equilibrium 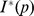 increases with *k* (i.e. the effectiveness of population reduction reduces) and when *k* is greater than some critical value *k_p_*, 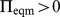. However, another behaviour is also apparent: when *k* approaches a lower threshold *k_t_*, there is a temporary increase in *I*(*t*), which results in 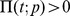 for a short period, despite no perturbation effect in the long term (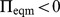). We call these two increases the persistent and the transient perturbation effect, and examine their properties in the following sections. Both persistent and transient perturbation effects are also possible in the case of emerging disease (when starting from close to the disease free equilibrium) (see Fig. 1B).

Behaviour in the long-term equilibrium can be seen by plotting the endemic equilibrium 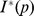 versus population reduction rate *p*, for several values of the horizontal transmission rate *β*. Three important points are evident (see Fig. 1C). First, persistent population reduction at a sufficiently intense rate does reduce the level of disease, leading to 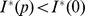. Second, the maximum size of the persistent perturbation effect reduces as the horizontal transmission rate increases, with no perturbation effect present in the deterministic model for *β* sufficiently high. Finally, increased horizontal transmission induced by population reduction can allow the disease to persist, where it would otherwise fade out in the absence of culling.

### Persistent perturbation effect with no disease induced mortality

We now explore the properties of the persistent perturbation effect 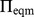 in more detail. For clarity we focus on the algebraically simpler case where there is no disease induced mortality, *e* = 0, and technical details of the analysis are given in Appendix S1.5 (in File S1). Subsequently we apply numerical analysis to Eqn. 1 with disease induced mortality *e*>0.

Case 1: Disease persists without population reduction, I*(0)>0

In this case there is a perturbation effect if
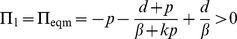



#### Minimum disease enhancement required to produce perturbation effect

Note that when *k* = 0, we obtain 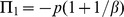, which is always negative, showing that culling reduces disease when there is no mechanism enhancing disease transmission. Rearranging gives a threshold value of *k*, above which a perturbation effect is possible
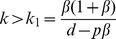
There is a lower bound on this threshold, such that 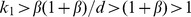 (since the disease is able to persist, which requires that 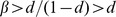, hence 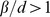).

#### High disease prevalence precludes a perturbation effect

**Figure 2:**
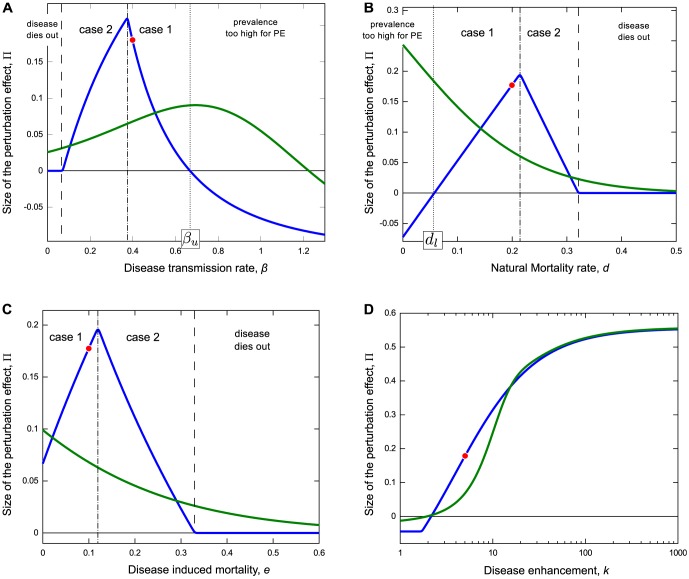
Sensitivity analysis of the persistent and transient perturbation effects in the deterministic model. Parameter values as in Table 2 and marked by a red dot when explicitly varied, the transient perturbation (green) is shown for *t* = 5, the persistent perturbation (blue) 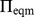 is evaluated at 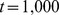. The transient perturbation: (A) has an optimum for intermediate *β*, decays with (B) natural and (C) disease induced mortality, and (D) increases with *k*. With the exception of (D), the behaviour of the persistent perturbation is more complex. In (A) to the left of 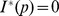 (dashed vertical line) *β* is low and there is no perturbation (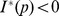). To the right of 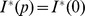 (dotted vertical line corresponding to upper bound 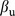, see text) the prevalence in the absence of culling is sufficiently high to prevent a perturbation. The central region between 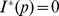 and 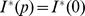 is divided by a third vertical line 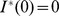 (dot dashed), independent of *k* and *p*, into regions corresponding to case 1 (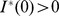) where the disease persists the absence of population reduction, and case 2 (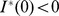) where it does not (see text for details). The maximum persistent perturbation occurs at this boundary. Under case 2, population reduction is sufficient to stabilise the endemic equilibrium. In (B) as natural mortality *d* increases from zero (moving left to right) 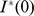 *decreases*, and the pattern seen in (A) is reversed. Here the dotted vertical line 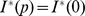 denotes the lower bound 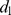. (C) shows the impact of disease induced mortality *e* is similar to that of natural mortality, but the chosen parameter values mean that prevalence is never too high to prevent a perturbation effect. Note: dotted and dashed lines are reversed when *k* is too low for the perturbation effect to occur, leaving no room for cases 1 and 2. See Fig. 4 for analogous spatial model results.

As 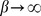, 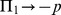, showing that for sufficiently high *β*, the perturbation effect cannot occur in the deterministic model. In fact in this case there is an upper bound, *β_u_*, on the value of *β* for which 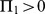,
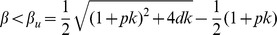
and 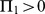 only when 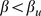 (see Fig. 2A for high *β*). Similarly, as *d*→0, 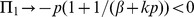, showing that the perturbation effect is possible only for higher mortality rates. There is a corresponding lower bound on *d* for which 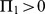, at
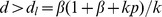
(see Fig. 2B, for low *d*). For low 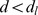, infectives are removed from the population slowly, and for high 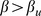, the disease spreads quickly; either situation leads to disease saturation, with insufficient susceptibles to allow for a perturbation effect.

#### High rates of population reduction will reduce disease levels

A simple observation is that a persistent perturbation effect is possible (for any model) only if the population size under persistent culling is greater than the equilibrium number of infected individuals without population reduction i.e. 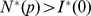 which implies that there is an upper bound on the culling rate, 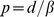, above which population reduction will reduce disease (see Appendix S1.5 in File S1). This is also evident in *k*_1_ (the lower bound for *k*) which diverges as 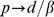 from below implying that, in order to see a perturbation effect, population reduction must produce ever greater enhanced transmission *k* as *p* approaches this critical level. Furthermore (see Appendix S1.5 in File S1), we show that the range of *p* that permits the perturbation effect also depends on *k* and is given by
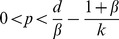
This is illustrated in Fig. 1C, where the range of *p* for which 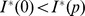 decreases with *β*, and that for sufficiently large *p*, 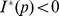 for all *β*.

Case 2: Disease does not persist without population reduction, I*(0)>0

#### Population reduction can allow disease to persist where it would naturally fade out

In this case there is a persistent perturbation effect if the disease is only able to persist under continued population reduction for a given *p* and *k*, i.e. when
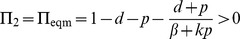
The conditions for which the disease is only able to persist under population reduction are detailed in Appendix S1.4 in File S1. The minimum *k* in order to make the endemic equilibrium 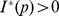 following population reduction is
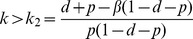
and therefore sufficiently large *k* can lead to a perturbation effect under these conditions. For example, given 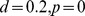, 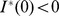 for 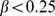; however, given 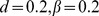, (thus unable to persist for 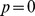), when population reduction is applied at rate 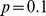, then 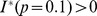 for 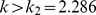, therefore the disease can persist as long as population reduction is sustained, leading to a perturbation effect (see Fig. 1C, for 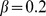).

### Persistent perturbation effect with disease induced mortality

We now investigate the persistent perturbation effect in the more complex situation with disease induced mortality *e*>0 by solving Eqn. 1 numerically to show how 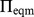 varies with *e* itself, and also with horizontal disease transmission *β*, background mortality *d*, and enhanced transmission *k* resulting from population reduction.

Numerical analysis of the role of transmission rate *β* is consistent with the analysis of the previous section (see Fig. 2A). Under case 1 (where 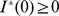 and 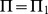), 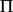 decreases with *β* and no perturbation is possible for 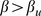 because the disease has saturated the population, whereas in case 2 (where 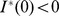 and 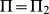), 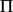 increases with *β*, and there is a lower limit below which the disease becomes extinct despite enhanced disease transmission. This is in accordance with analysis of 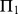 and 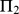 (see Appendix S1.5 in File S1, and above).

The role of natural mortality *d* is also consistent with the previous analysis (see Fig. 2B). In the region of case 1, there is a lower bound 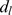, below which the perturbation effect is not possible due to disease saturation, and above which 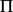 increases with *d*. In the region of case 2, 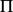 decreases with *d*, and there is an upper limit on *d*, above which the disease becomes unable to persist despite enhanced transmission. The role of disease induced mortality *e*, is broadly similar to that of *d* (see Fig. 2C).

The impact of the disease enhancement parameter *k* on the perturbation effect is illustrated for case 1 in Fig. 2D. 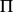 increases with *k*, tending to an asymptote as 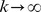, while there is no perturbation effect below the threshold 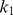. The behaviour under case 2 (not shown) is broadly similar with a different lower bound 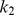 and lower asymptote.

### Maximising the persistent perturbation effect

An important addendum to these results is related to the conditions that maximise the perturbation effect. For low mortality rates *d* or *e*, or high transmission rate *β*, the disease is able to persist before and during population reduction, the prevalence is very high and there is little room for further increase. As mortality increases or transmission decreases, the size of the perturbation effect 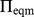 increases until 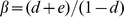, where the endemic equilibrium becomes negative, and the disease becomes unable to persist for 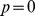 (as in case 2). After this point, as mortality increases, or transmission decreases, 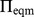 decreases, until mortality is too high, or transmission is too low to maintain the disease either before or during population reduction. This implies that the maximum perturbation effect occurs when 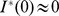 and 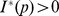. Therefore in practice, the persistent perturbation effect is most likely in a disease with very low prevalence. These results can be seen graphically in Fig. 2.

### Transient perturbation effect

The transient perturbation effect can be assessed by linearising the system and examining the rate of change of 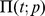 with respect to time, at time *t* = 0, which is positive (i.e. the disease increases faster under population reduction) only if 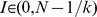 (see Appendix S2.1 in File S1). To obtain an initial increase in disease levels there must be some infectives, but similar to the results in the persistent case, too many infectives will prevent a transient perturbation effect; as *k* increases a transient perturbation effect is possible for ever larger numbers of infectives. The lower bound here is equivalent to 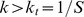 and since 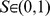, this requires that 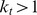; therefore, the transient perturbation effect does not occur in the absence of a change in behaviour. It is also possible to show that the transient perturbation effect increases fastest when 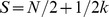 and 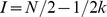 (i.e. roughly equal numbers of susceptibles and infectives) and that 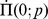 increases with both *p* and *k* (see Appendix S2 in File S1 for details). Also, a temporary peak, where 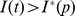 may occur, if the disease increases quickly before culling reduces the population size *N*; this can be observed in both endemic and emerging disease cases (see Fig. 1).

#### Starting from the endemic equilibrium

Consider the case where the disease is in the endemic equilibrium 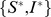 prior to disease intervention (as shown in Fig. 1A). We show in Appendix S2 (in File S1) that 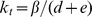 so that the minimum disease enhancement required for a transient perturbation effect is reduced when the infection rate *β* is small and mortality rates *d* and *e* are large. In addition 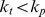 (where 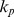 is the relevant 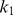 or 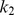) and the transient perturbation effect occurs for smaller *k* than the persistent perturbation effect. Consequently, for small 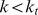, there is no perturbation effect. For larger 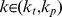, 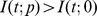 for small *t*, i.e. the number of infectives is initially larger following disease intervention, however eventually 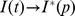 which is less than the initial level 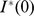, and in this case the increase is temporary. However, for 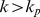, the number of infectives increases and remains higher than the control.

#### Starting from near the disease free equilibrium

The situation is somewhat different in the case of an emerging outbreak where 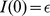 where 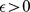 is small, and 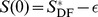, as shown in Fig. 1B (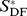 is *S* in the disease free equilibrium, see Appendix S1.3 in File S1). Here, 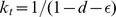 (see Appendix S2.2 in File S1), and so the minimum disease enhancement required for a transient perturbation effect is reduced when the initial prevalence is low (although contrary to the persistent perturbation effect, when mortality rates are also low). Fig. 2 shows the impact of varying *d*, *e*, *β* and *k* on the transient perturbation effect for the case of an emerging outbreak where 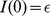 where 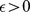 is small, and 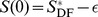. These numerical results show that the transient perturbation monotonically decreases with both natural and disease induced mortality, whilst it monotonically increases with enhanced transmission *k*. The disease transmission rate *β* affects the time disease takes to reach equilibrium, and therefore small *β* can result in a slow initial increase (and small transient perturbation effect), while very large *β* can saturate the population and prevent the transient perturbation effect from occurring at the time considered; the largest increase therefore occurs with an intermediate value of *β*, although this will vary depending on the time at which the transient perturbation effect is assessed.

These results contrast with those for the persistent perturbation effect (also shown in Fig. 2), demonstrating that conditions required for the transient and persistent perturbation effect are not necessarily the same for both emerging and endemic disease.

### Implicit enhancement of disease transmission induced by population reduction

We now show how the intrinsic dynamics of a natural spatial formulation of disease transmission and demography may give rise to an increased effective horizontal transmission when population reduction is applied, leading to an implicit perturbation effect. The non-spatial results of the previous section suggest that perturbation is strongest when disease prevalence is relatively low and where population reduction is intermediate, and gives rise to a sufficiently large increase in the horizontal transmission rate.

We begin by demonstrating the importance of heterogeneity in the model, and show through analysis of the horizontal infection rate in a simple two-site model that in the spatial model such an enhancement of the transmission rate will be strongest in situations where infection levels are most heterogeneous between groups.

### Heterogeneity and the perturbation effect in the spatial model

Consider a simple two-site model, with density dependent dispersal between the two groups A and B. The global infection rate *H* is
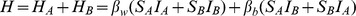
Assuming disease induced mortality rate *e* = 0, then 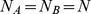, and the number of infectives is 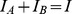, thus *H* can be simplified to
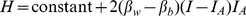
revealing that when between-group infection rate *β_b_* is small, and 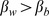, *H* is maximised when the infection is distributed evenly between sub-populations, and 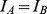. Conversely, *H* is minimised when 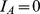 or 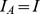 (i.e. all infectives are restricted to one of the groups).

In order to quantify enhanced transmission resulting from population reduction, consider the rate of change of the global infection rate *H* (differentiated with respect to time) to obtain
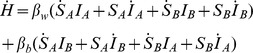
While 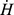 is affected by all processes, including birth, death, infection and dispersal, if we only examine the effect of dispersal on 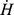 by substituting only the relevant components (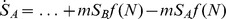 etc.) then we obtain
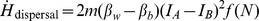
Consequently, if 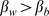 and 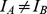, then the effect of dispersal is to increase 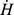. Moreover, this rate of increase in horizontal disease transmission is greatest for heterogeneously distributed disease (larger difference 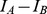), and for larger dispersal rate *m*. The presence of the density dependence function f(N) shows that it is greater for smaller *N*, which will follow as a consequence of population reduction. Note that the formula for *H* shows that a large *β_b_* will lead to rapid spread between sites, quickly spreading to sub-populations, and reducing spatial heterogeneity in the distribution of disease.

### Initial conditions

**Figure 3:**
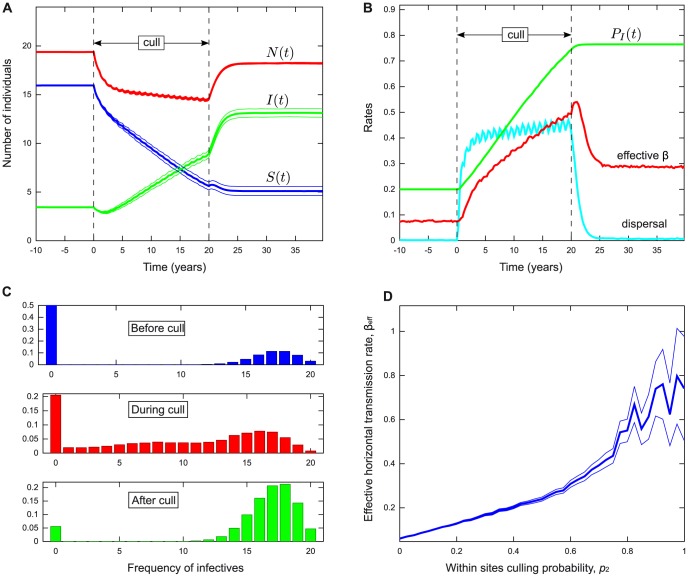
Time trajectories and heterogeneity for emergent disease in the stochastic model. (A) Population numbers, *S*(*t*), *I*(*t*), and *N*(*t*). (B) Proportion of sub-populations containing infectives, 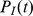, effective transmission rate *β*, and dispersal rate. (C) Distribution of *I* across sites. (D) Effective transmission rate *β* for disease transmission vs population reduction coverage *p*_1_. Parameters are given in Table 2, and initial conditions are at the disease free equilibrium 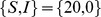, while in 20% of sites randomly chosen, a single individual is infected, resulting in 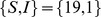. Population reduction occurs annually from years 50–69, and in 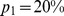 of sites (chosen randomly each year) the removal rate is set to 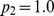, without regard to disease status (equivalent to an overall culling rate of 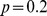). An initial reduction in *I* is rapidly replaced by an increase, which is due to the increased chance of invasion of naïve groups by infectives due to the density dependent dispersal. The CI for the effective transmission rate increases for large 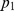 due to the increasing number of simulations where the disease becomes extinct.

Given the above discussion, when studying the spatial model we focus on cases where disease is distributed heterogeneously between groups and overall prevalence is low. This is most easily achieved when the system is close to the disease free equilibrium with: (i) disease maintained in each site by high within-site transmission rate *β_w_* and low mortality; (ii) low levels of disease transmission between sites; and (iii) relatively large and stable populations at each site leading to low levels of dispersal between sites. Under this scenario, even in the absence of population reduction, the number of sites infected, and thus overall prevalence, tends to slowly increase (from close to the disease free equilibrium) as rare dispersal or transmission events spread disease. Fig. 3 (discussed in detail below) shows how transient perturbation effects occur in such a system. In contrast, we show in Appendix S3.2 (in File S1) that by making both disease and population less stable within sites it is possible to achieve a dynamic quasi-equilibrium (quasi- because the ultimate fate of all simulations of this model is total extinction) where the spread of disease to uninfected sites is balanced by spontaneous recovery of infected sites, e.g. through death of infectives and birth of susceptible individuals. When the system is in such an endemic state population reduction leads to a persistent perturbation effect, as we saw in the non-spatial model (see Fig. S2 in File S1). However, this endemic state is very sensitive to the balance between site-level establishment and recovery of disease which makes it difficult to explore variation in the perturbation effect with respect to the value of key parameters. We therefore focus attention on the transient perturbation effect when starting close to the disease free state in the spatial model.

### Transient perturbation effect in the spatial model

The behaviour during population reduction in the spatial model is shown for the population values 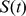, 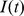, and 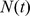 (see Fig. 3A), and the proportion of infected sites 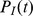, dispersal rates and effective transmission rate 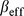 (see Fig. 3B). The distribution of infectives between sites is shown in Fig. 3C before, during, and after population reduction. Prior to population reduction, sites can be classified as disease-free or infected. During population reduction, the typical level of disease within sites decreases, but the number of infected sites increases. When population reduction ceases, typical prevalence in infected sites returns to previous levels which, given that there are now more of them, leads to a rapid increase in global prevalence. Some light may be shed on the mechanisms behind such changes, as population reduction leads to a large increase in dispersal, followed by increasing rates of horizontal disease transmission, *H* (see Fig. 3B). Population reduction disrupts the stable demographic structure (shown in Fig. 3C) leading to an increase in the dispersal rate and movement of infectives to previously disease free sites. This *vacuum effect* [5], [26] emerges from the spatial model's density dependent dispersal and leads to increased transmission. The effective horizontal transmission rate parameter varies with population reduction effort *p*_1_: for small *p*_1_, there is an almost linear increase in *β*_eff_ (see Fig. 3D), which agrees well with the explicit increase assumed to be 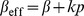 in the non-spatial model. However, one key difference (as shown in Fig. 3B), is that the increase is not immediate, but grows linearly with time — an effect not accounted for by our earlier analysis.

**Figure 4:**
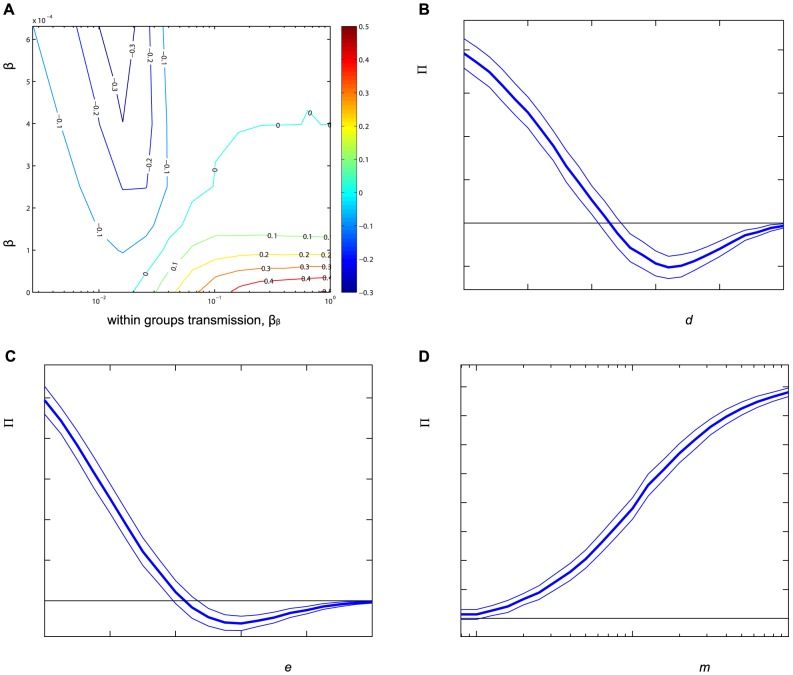
Sensitivity analysis of 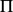 in the stochastic model. The size of the perturbation effect, 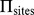, at time 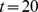 starting near the disease free equilibrium for (A) Between and within-groups infection rates 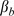 and 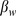 (log scale). (B) Natural mortality rate *d*. (C) Disease induced mortality rate *e*. (D) Dispersal rate *m* (log scale). Default parameters are given in Table 2, and one parameter is varied at a time. This is analogous to Fig. 2 for the non-spatial case. Initial conditions are such that 20% of sites are randomly chosen to start near the endemic equilibrium (with a minimum of 1 infective), while the remainder begin at the disease free equilibrium.

We now explore the sensitivity of this perturbation effect with respect to key aspects of demography and disease dynamics. The results are broadly consistent with those obtained when starting close to the disease free equilibrium in the non-spatial model. Fig. 4 shows results for parameters analogous to those in Fig. 2, and that 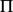 decreases with mortality rates *d* and *e* and increases with dispersal rate *m* (similar to *k* in the non-spatial case). The role of disease transmission is more complex. The perturbation effect decreases with between-group infection rate 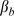 which reduces the number of disease free sites, and increases with 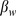, which increases disease persistence within sites. Thus for small 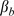 and sufficient 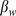, population reduction is able to spread disease to uninfected sites where it is able to persist. We also explore the impact of varying the threshold parameter *α* that determines how sensitive the rate of dispersal is to local reductions in the size of the population in the destination site (see Appendix S3.1 and Fig. S1 in File S1). Results show that a perturbation effect occurs for a wide range of values, although the largest effects are seen for *α* around 0.9 (we suspect the largest increase would be observed for *α* near 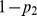). Perturbation effects were also found for alternative forms of the density dependent dispersal function 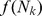 (results not shown).

## Discussion

In this paper we explored the impact on disease control of enhanced transmission resulting from individual or demographic responses to population reduction. Using a generic non-spatial and deterministic model of demography and disease dynamics we explored the potential for such effects to reduce and reverse the disease control benefits of population reduction. We found that there was a threshold of enhanced transmission above which a perturbation effect occurred, whereby the number of infected individuals increases during the period when population reduction is applied. However, sufficient population reduction (the level rising with mortality rates *d* and *e* and disease enhancement *k*, but decreasing with infection rate *β*) will always reduce numbers of infectives in the area it is applied. Disease systems with low levels of disease are more sensitive to the impacts of enhanced transmission. For systems with endemic disease, the potential for the perturbation effect increases with natural and disease induced mortality rates (due to reduced levels of endemic disease), with the opposite trend where disease is emerging, as higher mortality removes cases caused by enhanced transmission. With respect to the horizontal transmission rate, the perturbation effect in endemic disease is maximised for small to intermediate *β* (at the point where the disease changes its ability to persist in the absence of population reduction). For emerging disease however, higher *β* causes the disease to reach equilibrium sooner, but reduces the size of the perturbation effect, so the earlier the disease is measured, the higher the optimal *β*, but the weaker the perturbation. Enhanced transmission effects can also lead to disease being maintained by population reduction in systems where it would otherwise die out.

We also considered a spatially explicit model that represents demographic fluctuations and disease transmission within locally well mixed populations, and dispersal and disease transmission between such groups. In this context we found that enhanced transmission emerged implicitly as a demographic response to population reduction when dispersal was density dependent. This enhancement would be increased if individuals explicitly changed their behaviour, e.g. by dispersing more or by increasing agonistic interactions and therefore disease contacts between groups (i.e. increasing *β_b_*). However, the implicit dispersal mechanism alone was sufficient to give rise to a perturbation effect. We found that the system was susceptible to enhanced transmission in both the case of endemic and emerging diseases when infection was heterogeneously distributed among groups and when overall levels of disease were relatively low. For emerging disease we showed that the impact of mortality rates was qualitatively similar to the predictions of the non-spatial analysis. In the spatial model, dispersal rate played a similar role to the non-spatial enhancement parameter *k*, whereas the role of horizontal disease transmission is not directly comparable between the two cases. In the spatial context, higher within-group transmission increased the size of the perturbation effect, but even low rates of between-group transmission reduced it. Analysis of the effective contact rate in the spatial model reveals that enhanced transmission varied in time and this could be incorporated in future analysis of the non-spatial system. It is worth noting that the linear assumption *kp* for disease enhancement is reasonable, at least early in disease intervention period, and for low removal rate *p*.

Many authors have noted problems related to disease control via population reduction in wildlife [10], [21], [22], [24], [32], including situations were disease risks are increased rather than reduced [13], [25]. Individual behavioural [18]–[20] and demographic [33] responses to population reduction are thought to enhance disease transmission in wildlife. One system of particular relevance is TB in badgers, where the disease is spatially heterogeneous [34], transmission between groups is weak, and the host exhibits density dependent dispersal [29], [30], [35]. Thus TB in badgers is a disease system exhibiting many of the properties this paper shows are likely to lead to the perturbation effect, and we note that the RBCT [23] did indeed show that culling was associated with an increase in bTB prevalence in badgers [25]. Moreover, the results of this paper suggest that a wide range of wildlife disease systems are sensitive to such effects, and this is consistent with the marked inefficiencies of population reduction as a disease control strategy observed to date. However, the effects studied here are likely to be even more widespread than current empirical studies suggest as they undermine the efficacy of population reduction measures even in situations where they do not lead to a complete reversal of its effectiveness.

## Supporting Information

File S1 (PDF)
