# Supplementary material for: Demographic Processes Drive Increases in Wildlife Disease following Population Reduction
Source: PLoS One. 2014 May 2;9(5):e86563. doi: 10.1371/journal.pone.0086563 (PMC4008369; doi:10.1371/journal.pone.0086563)
Supplement: File S1 — (PDF) [file pone.0086563.s001.pdf]

Demographic processes drive increases in wildlife disease following population reduction — Supporting Information

Jamie C. Prentice[1,2,3], Glenn Marion[2,*], Piran C.L. White[3], Ross S. Davidson[1], Michael R. Hutchings[1]
1 Disease Systems Team, SRUC, Edinburgh, United Kingdom
2 Biomathematics and Statistics Scotland, Edinburgh, United Kingdom
3 Environment Department, University of York, York, United Kingdom
∗ Corresponding author E-mail: glenn@bioss.ac.uk

Appendix S1

Derivation and analysis of the deterministic non-spatial model

Appendix S1.1 Formulation of non-spatial deterministic model

A basic deterministic SI model for disease [1], without population reduction, can be written as:

$$\frac{dS}{dt} = rN(1 - N/c) - dS - \beta SI$$
$$\frac{dI}{dt} = -(d + e)I + \beta SI$$

The first equation represents the rate of change of the susceptible population in terms of three processes: birth of susceptibles at rate $rN(1 - N/c)$ (note all individuals are assumed susceptible at birth since we ignore vertical and pseudo-vertical transmission); death of susceptibles at rate dS and infection of susceptibles at rate βSI. Similarly, the second equation represents the dynamics of the infected population which increases as susceptibles become infected at rate βSI, and decreases as infectives die due to the effects of the disease at rate eI and due to other natural causes at rate dI.

As described in the main text we model the effect of simple population reduction by introducing an additional death rate p. Thus the death rates become $(d + p)S$ and $(d + e + p)I$ for susceptibles and infectives respectively. To account for the impact of changes in host susceptibility and behaviour induced by population reduction we add kp to the horizontal disease transmission rate which therefore becomes $(\beta + kp)SI$. The resulting equations are

$$\frac{dS}{dt} = rN(1 - N/c) - (d + p)S - (\beta + kp)SI$$
$$\frac{dI}{dt} = -(d + e + p)I + (\beta + kp)SI$$

Appendix S1.2 Rescaling the model

It is possible to simplify the above equations, by scaling the variables representing population sizes S, I, and N, and time t to remove two of the parameters. Here we choose to rescale the population sizes by $1/c$,

$$\hat{S} = S/c, \quad \hat{I} = I/c, \quad \hat{N} = N/c$$

which transforms the values from $N \in [0, c]$ to $\hat{N} \in [0, 1]$. We choose also to rescale time by r, since r is the least interesting parameter to examine.

By subtituting the rescaled variables into the model, the remaining parameters can be obtained:

$$\hat{d} = d/r, \quad \hat{e} = e/r, \quad \hat{p} = p/r, \quad \hat{\beta} = \beta c/r, \quad \hat{k} = kcr \tag{S1}$$

which leads to Eqn. 1 in the main text (dropping the hat notation for convenience). Unscaled solutions can then be obtained by back scaling by c and r appropriately, for example if the scaled value of $\hat{S}^*_{\mathrm{DF}} = 1 - \hat{d}$, then the unscaled value is $S^*_{\mathrm{DF}} = c(1 - d/r)$.

Appendix S1.3 Disease free and endemic equilibria

Solving Eqn. 1 for biologically realistic steady states, where $\dot{S} = \dot{I} = 0$, and $S, I \geq 0$, gives:

1. *Population extinction*, at $S^*_0 = 0$, $I^*_0 = 0$.
2. The *disease free equilibrium*, at $S^*_{\mathrm{DF}} = 1 - d - p$, $I^*_{\mathrm{DF}} = 0$. S^*_{DF} is often referred to as the population equilibrium, K.
3. The *endemic equilibrium*, at

$$S^* = \frac{d+e+p}{\beta + kp},$$

$$I^* = \frac{1}{2}(1-d-e-p) - \frac{d+e+p}{\beta+kp} + \frac{1}{2}\sqrt{(1-d-e-p)^2 + 4\frac{d+e+p}{\beta+kp}e}$$

When $e = 0$, provided that $d + p < 1$ (i.e. where the population persists because the birth rate, rescaled to 1, exceeds the combined mortality and removal rate), the endemic equilibrium simplifies to

$$S^* = \frac{d+p}{\beta+kp}, \quad I^* = 1 - d - p - \frac{d+p}{\beta+kp}$$

and when $p = 0$, the endemic equilibrium simplifies further to

$$S^* = d/\beta, \quad I^* = 1 - d - d/\beta$$

Appendix S1.4 Disease stability

Equilibria are stable (i.e. attract trajectories) when all eigenvalues have negative real parts, unstable (i.e. repel trajectories) when all eigenvalues have positive real parts, or a saddle node when some are positive and the rest are negative (e.g. this may allow the disease free equilibrium to be stable in the absence of infectives, but unstable in their presence). Note that while an equilibrium can be mathematically negative, it directs a biological process, which must remain non-negative, and thus a negative equilibrium cannot be reached. We examine the eigenvalues of Eqn. 1 (main text), with $e = 0$ for tractability. These are:

$$\lambda_1 = 1 - d - p - 2N, \quad \lambda_2 = (\beta + kp)(S - I) - d - p$$

Evaluating the eigenvalues for the endemic equilibrium gives:

$$\lambda_1 = d + p - 1, \quad \lambda_2 = -(\beta + kp)(1 - d - p) + d + p$$

Evaluating the eigenvalues for the disease free equilibrium gives:

$$\lambda_1 = d + p - 1, \quad \lambda_2 = (\beta + kp)(1 - d - p) - d - p$$

$\lambda_1 < 0$ if $d + p < 1$ (required for non-population extinction), therefore the endemic equilibrium is stable, and the disease free equilibrium is a saddle node if $\lambda_2 < 0$, which occurs when:

$$\beta > \frac{d+p}{1-d-p} - kp$$

otherwise the stabilities are reversed. For $p = 0$, this becomes $\beta > d/(1-d)$.

Assuming the endemic equilibrium is stable, it can only persist if $I^* > 0$. This is equivalent to $S^* < S^*_{\mathrm{DF}}$, provided that $e \geq 0$, which is an easier calculation. Therefore $I^* > 0$ if

$$\frac{d+e+p}{\beta+kp} < 1-d-p$$

Bounds under which the disease can persist may be obtained for each of the parameters by rearranging the above inequality as follows:

$$\begin{aligned}
d &< \frac{(1-p)(\beta+kp)-e-p}{1+\beta+kp} \qquad \text{(S2)}\\
e &< (\beta+kp)(1-d-p)-d-p \\
\beta &> \frac{d+e+p}{1-d-p}-kp \\
k &> \frac{d+e+p}{p(1-d-p)}-\frac{\beta}{p} \\
p &< \frac{1}{2}\left(1-d-\frac{1+\beta}{k}\right)+\frac{1}{2}\sqrt{\left(1-d-\frac{1+\beta}{k}\right)^2-4\frac{d+e-(1-d)\beta}{k}}
\end{aligned}$$

In the absence of population reduction, when $p = 0$ these become:

$$\begin{aligned}
d &< \frac{\beta-e}{1+\beta} \\
e &< \beta-d-d\beta \\
\beta &> \frac{d+e}{1-d}
\end{aligned}$$

Appendix S1.5 Conditions for the deterministic perturbation effect $\Pi_{\mathrm{eqm}} > 0$

The size of the persistent PE is given by:

$$\Pi_{\mathrm{eqm}}(p) = \max\{I^*(p), 0\} - \max\{I^*(0), 0\}$$

Focussing on the algebraically tractable case without disease induced mortality, $e = 0$, and on situations where disease is still present with population reduction, $I^*(p) > 0$, we examine two cases:

Case 1: $I^*(0) \geq 0$, whence

$$\Pi_{\mathrm{eqm}} = \Pi_1 = -p - \frac{d+p}{\beta+kp} + \frac{d}{\beta}$$

Case 2: $I^*(0) \leq 0$, whence

$$\Pi_{\mathrm{eqm}} = \Pi_2 = 1-d-p-\frac{d+p}{\beta+kp}$$

Note that $\Pi_2 = S^*_{\mathrm{DF}} - S^*$, and so for case 2, the values are exactly the same as for Eqns. S2 in Section Appendix S1.4. Also note that $\Pi_1 = \Pi_2$ when the stability changes, i.e. when $I^*(0) = 0$.

Next, we look for conditions on the perturbation effect under Case 1, by solving $\Pi_1 > 0$ for different parameters (conditions for Case 2 correspond to the bounds for stability given by Eqns. S2).

- Natural mortality rate d. Rearranging $\Pi_1 > 0$ for d, we obtain:

$$d > \beta(1 + \beta + kp)/k$$

- Infection rate β. Similarly:

$$\beta < -\frac{1}{2}(1 + kp) + \frac{1}{2}\sqrt{(1 + kp)^2 + 4dk}$$

- Population reduction rate p. First, observe that if the number of infectives prior to population reduction is greater than the total population size under persistent population reduction, then there is no room for I to increase. Therefore

$$\begin{aligned} I^*(0) &< N^*(p) \\ \Rightarrow p &< d/\beta \end{aligned} \tag{S3}$$

  However, rearranging $\Pi_1 > 0$ gives:

$$0 < p < \frac{d}{\beta} - \frac{1 + \beta}{k} \tag{S4}$$

  For which Eqn. S3 is the limit as $k \to \infty$.

- Behaviour change k. We look for conditions on the perturbation effect under both cases:

  Case 1:

$$k > k_1 = \frac{\beta(1 - \beta)}{d - \beta p}$$

  Case 2:

$$k > k_2 = \frac{d + p - \beta(1 - d - p)}{p(1 - d - p)}$$

  Note that k_2 is the minimum k for which the disease is able to persist(i.e. population reduction maintains the disease when $k > k_2$). Also note that $k_1 = k_2$ when $I^*(0) = 0$.

This demonstrates that β is bounded below in order for the disease to persist, $0 < I^*(p)$, however it also bounded above in order for the disease to increase with population reduction, $I^*(0) < I^*(p)$. If both of these bounds are satisfied, then $0 < I^*(0) < I^*(p)$ (which gives Case 1). Note that this interval increases with k.

Appendix S2

Transient perturbation effect

Appendix S2.1 Analysis

While intermediate behaviour of $I(t)$ prior to the disease reaching a new equilibrium can only be solved numerically, both the long term and initial behaviour can be analysed algebraically. Here we examine the initial behaviour of $\Pi(t; p)$ by linearising the system, and measuring the difference between $\dot{I}(t; p)$ and $\dot{I}(t; 0)$ at time $t = 0$. Substituting $I(t)$ from Eqn. 1 into Eqn. 3 (see main text), and differentiating with respect to time gives

$$\dot{\Pi} = \dot{I}(p) - \dot{I}(0) = pI(kS - 1)$$

hence $\dot{\Pi} > 0$ if

$$k > k_t = 1/S$$

Note that since $S \in (0,1)$, this requires that $k_t > 1$, therefore the transient perturbation effect does not occur in the absence of a change in behaviour.

It is immediately clear that the transient perturbation effect depends directly on, and increases with, p and k, however it also depends on initial conditions $S(0)$ and $I(0)$, which in turn depend on the remaining parameters. Intermediate behaviour must be found numerically, but some further insight can be obtained by observing how $\dot{\Pi}(t)$ depends on $S(t)$ and $I(t)$.

Substituting $S = N - I$ into $\dot{\Pi}$ gives a quadratic in I, which can be solved to show that $\dot{\Pi}_0 > 0$ when $I \in (0, N - 1/k)$, and is maximised when $S = N/2 + 1/2k$ and $I = N/2 - 1/2k$. Therefore the transient perturbation effect increases fastest when S and I are near these values (i.e. roughly equal), and larger k permits a greater range of I for which the transient perturbation effect is possible.

Appendix S2.2 Initial conditions

Endemic disease
We first examine the case where the disease is in the endemic equilibrium, $\{S^*, I^*\}$ prior to disease intervention. We substitute $S(0) = S^*$ into k_t, to obtain $k_t = \beta/(d+e)$, therefore the minimum behaviour change required for the perturbation effect is reduced when the infection rate β is small and mortality rates d and e are large. Note that

$$k_p = \frac{\beta(1+\beta)}{d - p\beta} > \frac{\beta(1+\beta)}{d+e} > \frac{\beta}{d+e} = k_t$$

which leaves $k_t < k_p$, so the transient perturbation effect occurs for smaller k than the persistent perturbation effect.
Emergent disease
If the disease is not in equilibrium prior to disease intervention, then another sensible initial condition to examine is when the disease has been newly introduced, in which case $I(0) = \epsilon$ where $\epsilon > 0$ is small, and $S(0) = 1 - d - \epsilon$, in this case the transient perturbation effect occurs if $k > k_t = 1/(1 - d - \epsilon)$, and so the minimum behaviour change required is when $I(0)$ is smallest, which agrees with the concept that the perturbation effect occurs most readily in diseases with low prevalence (as it does in the persistent case).

Appendix S2.3 Intermediate behaviour

Some insight into the intermediate behaviour can be obtained by noting that $\dot{\Pi}$ is a quadratic in I, maximal at $I = N/2 - 1/2k$, and negative for $I > N - 1/k$. This means that the perturbation effect will begin slowly when $I(0)$ is small, and then increase in rate as $I(t)$ passes through $N(t)/2 - 1/2k$. If $I > N - 1/k$, then $\dot{\Pi}$ becomes negative, and Π will decrease.

However, $N^*(p) < N^*(0)$ (since population reduction reduces the population size N), so the boundary $N - 1/k$ will decrease with time. This means that $\Pi(t)$ may increase for larger I near the start of the cull when N is larger than it can later on when N is smaller. If the boundary decreases below I, then $\dot{\Pi}$ becomes negative, forcing Π to decrease until it reaches the equilibrium value. Consequently it is possible for the disease to temporarily increase above $I^*(p)$, provided it happens early when the population has not yet been fully reduced in size (this explains the temporary peak seen near the start of the perturbation effect in Figs. 1A and 1B).

Appendix S3

Analysis and simulation of the perturbation effect in the stochastic spatial model

Appendix S3.1 Robustness of transient perturbation effect in spatial model

We examine the impact of varying certain mechanisms on the transient perturbation effect; in particular, the density dependent dispersal. In the main body we considered the case where $f(N_j) = 1$ when $N_j < \alpha N^*_{\mathrm{DF}}$ (where N^*_{DF} is the disease free equilibrium), and 0 otherwise.

The threshold parameter $\alpha = 0.7$ was arbitrarily chosen, however Fig. S1 shows that the transient perturbation effect occurs for a range of α that determines how sensitive the rate of dispersal is to local reductions in the size of the population in the destination site.

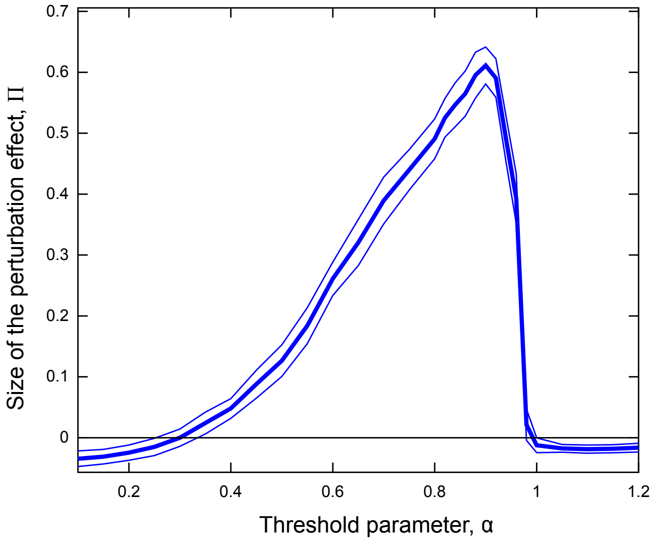

Figure S1. Sensitivity analysis of Π in the stochastic model. The size of the perturbation effect, Π_{sites}, at time $t = 20$ starting near the disease free equilibrium for dispesal threshold α. Default parameters are given in Table 1, and one parameter is varied at a time. Initial conditions are such that 20% of sites are randomly chosen to start near the endemic equilibrium (with a minimum of 1 infective), while the remainder begin at the disease free equilibrium.

Another, more linear, density dependent dispersal function, $f(N_j) = 1 - N_j/c$, was also explored. For this function, dispersal rates prior to population reduction tended to be much greater, and consequently the perturbation effect was much weaker (although still present); the strongest perturbation effect tended to occur when the disease free equilibrium, N^*_{DF}, was close to the carrying capacity c (i.e. when d is small), giving the greatest potential for increase in dispersal rates. Results (not shown), while weaker, were broadly similar to those found with the step function for density dependence function.

Appendix S3.2 Persistent perturbation effect in the spatial model

It is important to demonstrate that the persistent perturbation effect still occurs in the spatial model, however for this we require that the equilibrium value P^*_I remains between 0 and 1 (ideally near 0.5).

In addition, $P_I(t)$ should be allowed to fluctuate freely, with an non-negligible chance of the disease becoming extinct within any individual sub-population, and of the disease transferring to neighbouring disease free groups; thus, from any set of initial conditions, the system should reach equilibrium within a reasonable time period. Only a very narrow parameter range allows for this situation, while still allowing the perturbation effect to occur.

A set of parameters that allowed the perturbation effect to occur, while still allowing $P_I(t)$ to fluctuate freely and that $P_I(t) \to P_I^* \in (0,1)$ in a reasonable time period are: $c = 20$, $r = 1$, $d = 0.08$, $e = 0.46$, $m = 0.1$, $\alpha = 0.7$, $\beta_w = 0.8$, $\beta_b = 0$. See Fig. S2 for results.

References

1. Anderson RM, May RM (1979) Population biology of infectious diseases: Part I. Nature 280: 361–367.

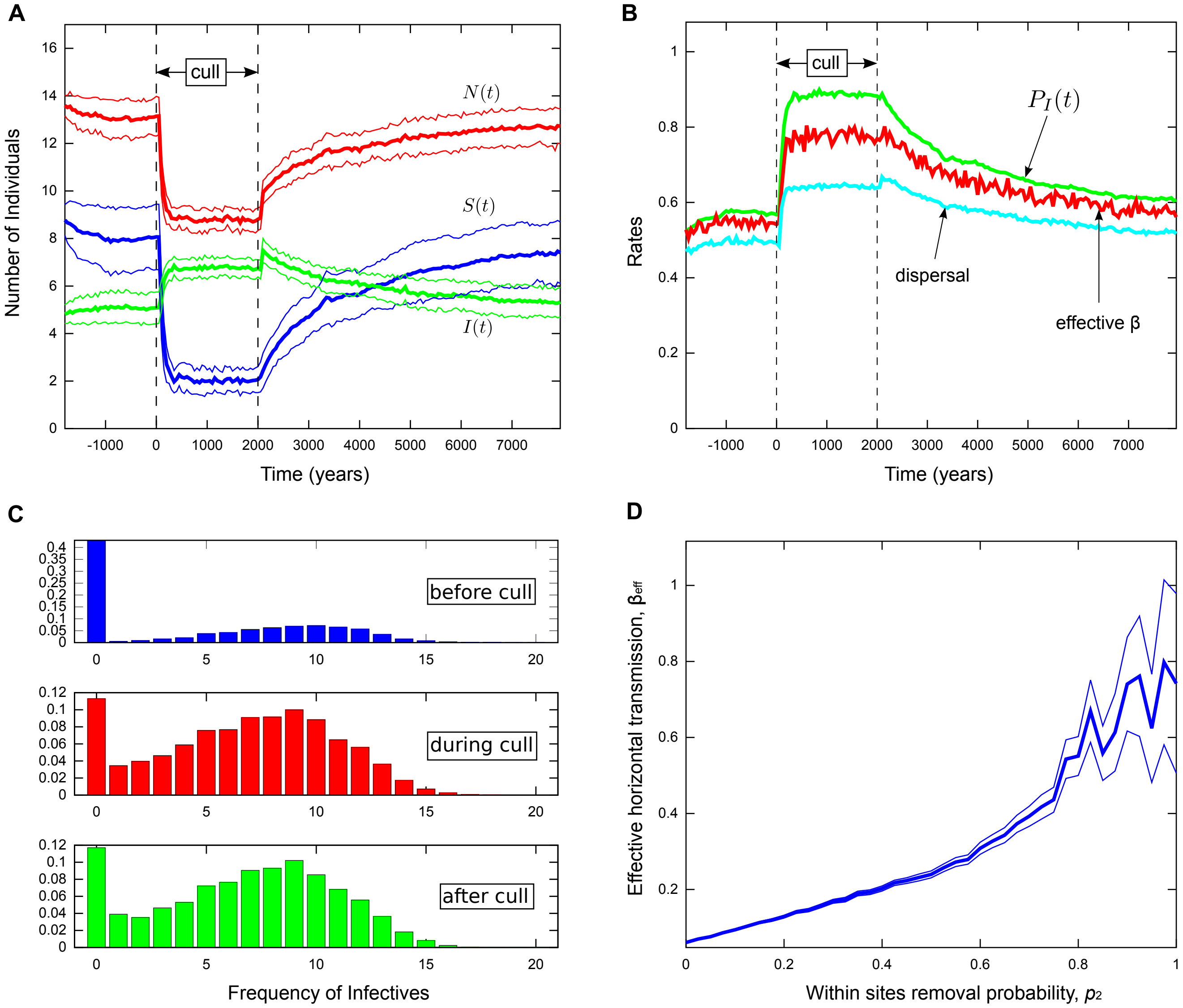

Figure S2. Time trajectories and heterogeneity for endemic disease in the stochastic model (A) Population numbers, $S(t)$, $I(t)$, and $N(t)$. (B) Proportion of sub-populations containing infectives, $P_I(t)$, effective transmission rate β, and dispersal rate. (C) Distribution of I across sites. (D) Effective transmission rate β for disease transmission vs population reduction coverage p_1. Parameters are: $c = 20$, $r = 1$, $d = 0.08$, $e = 0.46$, $m = 0.1$, $\alpha = 0.7$, $\beta_w = 0.8$, $\beta_b = 0$, $p_1 = 0.2$, $p_2 = 1.0$. These parameters were chosen to provide an equilibrium where $P_I(t)$ fluctuates, but remains roughly stable and between 0 and 1, and the perturbation effect still occurs. The system is given 2000 years to stabilise, then population reduction is applied for 2000 years, during which a new equilibrium appears to be reached. Afterwards, the system eventually returns to the original equilibrium.
